# Human articular chondrocytes express ChemR23 and chemerin; ChemR23 promotes inflammatory signalling upon binding the ligand chemerin^21-157^

**DOI:** 10.1186/ar3215

**Published:** 2010-12-30

**Authors:** Vivian Berg, Baldur Sveinbjörnsson, Signy Bendiksen, Jan Brox, Khaled Meknas, Yngve Figenschau

**Affiliations:** 1 Department of Laboratory Medicine, University Hospital of North Norway, Sykehusveien 38, N-9038, Tromsø, Norway; 2 Division of Immunology, Institute of Medical Biology, Faculty of Health Sciences, University of Tromsø, Sykehusveien 44, N-9037, Tromsø, Norway; 3 Childhood Cancer Research Unit, Karolinska University Hospital, Solna, S-17176, Stockholm, Sweden; 4 GenOk-Center for Biosafety, Science Park, Sykehusveien 23, N-9019, Tromsø, Norway; 5 Department of Medical Biochemistry, Institute of Medical Biology, Faculty of Health Sciences, Sykehusveien 44, University of Tromsø, N-9037, Tromsø, Norway; 6 Department of Orthopaedics, University Hospital of North Norway, Sykehusveien 38, N-9038, Tromsø, Norway

## Abstract

**Introduction:**

Chemerin is a chemotactic peptide which directs leukocytes expressing the chemokine-like receptor ChemR23 towards sites of inflammation. ChemR23 is a G protein-coupled receptor which binds several different ligands, and it is also expressed by other cell types such as adipocytes. In addition to chemotaxis, recent reports suggest that ChemR23 is capable of mediating either inflammatory or anti-inflammatory effects, depending on the type of ligand it binds. In the present study, we aimed to clarify whether human chondrocytes express ChemR23 and chemerin, and whether chemerin/ChemR23 signalling could affect secretion of inflammatory mediators.

**Methods:**

Tissue sections were taken from human knee joints and labelled with antibodies towards chemerin and ChemR23. Chondrocytes from cartilage tissue were isolated, cultured and assessed for chemerin and ChemR23 expression by PCR and immunolabelling. Receptor activation and intracellular signalling were studied by assessment of phosphorylated mitogen activated protein kinases (MAPKs) and phosphorylated Akt after stimulating cells with recombinant chemerin^21-157^. Biological effects of chemerin^21-157 ^were investigated by measuring secretion of pro-inflammatory cytokines and metalloproteases in cell supernatants.

**Results:**

Both serially cultured human articular chondrocytes and resident cells in native cartilage expressed chemerin and ChemR23. Stimulating cells with chemerin^21-157 ^resulted in phosphorylation of p44/p42 MAPKs (ERK 1/2) and Akt (Ser 473). Also, significantly enhanced levels of the pro-inflammatory cytokines interleukin-6 (IL-6), interleukin-8 (IL-8), tumour necrosis factor alpha (TNF-α), interleukin-1 beta (IL-1β), and the matrix metalloproteases MMP-1, MMP-2, MMP-3, MMP-8 and MMP-13 were detected.

**Conclusions:**

These results demonstrate that human chondrocytes express both the receptor ChemR23 and the ligand chemerin. Chemerin^21-157 ^stimulation engaged signal-transduction pathways that further promoted inflammatory signalling in chondrocytes, as judged by an enhanced secretion of cytokines and metalloproteases. Taken together, the previously reported chemotaxis and the present findings suggest that the receptor and its ligand may play pivotal roles in joint inflammation.

## Introduction

Migration of leukocytes to sites of inflammation is a hallmark of acute and chronic inflammation, and preventing cell recruitment to inflamed tissues is evidently a favourable strategy to reduce inflammation in arthritis [1]. Recognizing that chondrocytes mediate inflammatory signalling probably preceding leukocyte migration as in arthritis, these cells appear to be key actors in the early phase of the disease. Hence, it is importunate to clarify whether these cells express receptors that mediate pro-inflammatory signalling.

Chemerin, also known as tazarotene-induced gene 2 (TIG2), is a chemotactic peptide that binds the G protein-coupled receptor ChemR23 [2]. Chemerin has been detected at high levels in tissues such as psoriatic skin [3], in synovial fluid from arthritic joints and in ascitic fluids from human ovarian cancer and liver cancer [4,5]. Under normal physiological conditions, chemerin circulates in an inactive form as prochemerin at nanomolar concentrations, whereas activation is enabled by the proteolytic removal of amino acids at the C-terminal end by proteases of the coagulation, fibrinolytic and inflammatory cascades [6]. Prochemerin, which constitutes 143 amino acids, is a precursor for several isoforms of chemerin, including that in hemofiltrate and ascites identified as the isoform chemerin^21-157 ^[1]. In addition to chemotaxis, and by signalling through the receptor ChemR23, the isoforms produced by serine proteases possess pro-inflammatory properties, whereas those generated by cysteine proteases exert anti-inflammatory activities [1,7]. Accordingly, prochemerin appears to mediate dual effects, depending on the type of chemerin isoform produced.

The receptor ChemR23, also known as chemokine-like receptor 1 (CMKLR1), is expressed primarily by professional antigen-presenting cells such as dendritic cells (DCs) [5], natural killer cells and macrophages [8]. Hence, it is a leukocyte chemoattractant receptor which directs the migration of these cells to sites of inflammation. Neutrophils, the predominant leukocytes present during early acute inflammation, are capable of promoting maturation of prochemerin to chemerin, thus suggesting that the chemerin/ChemR23 signalling system may serve as a bridge between innate and adaptive immunity [1], as shown by the fact that ChemR23 is expressed by both myeloid DCs and plasmacytoid DCs, subsequently promoting adaptive immunity [9].

There is compelling evidence of beneficial effects of dietary supplements of eicosapentaenoic acid (EPA) in a wide range of human inflammatory conditions including arthritis [10-12]. The mechanisms explaining the beneficial effects of EPA is still debated, and the primary theory is that EPA interferes with the oxidation of aracidonic acid (AA), by competitive inhibition [10]. It has also been suggested that 15-lipoxygenase products of EPA can affect the transcription factor NF-κB, preventing the activation of inflammatory genes [13,14]. One interesting finding is that ChemR23 binds the endogenous lipid mediator derived from EPA, resolvin E1 (RvE1), that in leukocytes leads to anti-inflammatory signalling and promotion of resolution [13].

In the present study we aimed to clarify whether human articular chondrocytes express ChemR23 and whether recombinant chemerin^21-157 ^could elicit inflammatory signalling in these cells. Moreover, cellular expression of chemerin was investigated to unravel a possible inflammatory circuit in joints which may be exploited by lipidmediators derived from EPA to promote resolution.

## Materials and methods

The experiments were performed in accordance with The Code of Ethics of the World Medical Association (Declaration of Helsinki) for experiments involving humans. Patients gave a written informed consent to use biopsies for scientific purposes, and the project was approved by The Regional Ethics Committee.

### Acquisition of chondrocytes

Human articular chondrocytes from knee joints were obtained from patients subjected to autologous chondrocyte transplantation (ACT) and from osteoarthritic (OA) patients subjected to total knee arthroplasty. Biopsies from ACT patients were collected and prepared as previously described [15], while biopsies from osteoarthritic joints were taken from areas macroscopically judged as the healthiest part of the cartilage. In both cases, cells were isolated and cultured as previously described [16]. Briefly, cells were cultivated in growth medium Dulbecco's Modified Eagle's Medium (DMEM)/Ham's F12 (Cat. No. F4815, VWR, Oslo, Norway) supplemented with L-glutamine (Cat. No. K0302, VWR) gentamicin (Cat. No. G-1397, Sigma Aldrich, St. Louis, MO, USA) amphotericin B (Cat. No. A-2942, Sigma Aldrich) and 10% fetal calf serum (FCS) (Cat. No. N-4637, Sigma Aldrich). ACT cells were initially cultured in autologous human serum until transplantation, that is, three to four weeks, and thereafter cryopreserved. Subsequent propagation was supported by 10% FCS, whereas cells from osteoarthritic joints had growth medium supplemented with 10% FCS only.

Biopsies of cartilage serving as healthy controls were taken from patients subjected to surgery due to reconstruction of the anterior cruciate ligament (ACL). These patients were under the age of 35 and had no previous clinical symptoms of arthritis. Tissue removed as part of the surgical procedure was included in the study provided that it had no macroscopic signs of inflammation.

### Reverse transcriptase polymerase chain reaction (RT-PCR)

Messenger RNA (mRNA) from cultivated chondrocytes was extracted with Qiagen Direct mRNA kit (Merck Eurolab, Oslo, Norway). cDNA was synthesised by using SuperScript Preamplification System (Life Technologies Ltd., Paisley, UK) and treated with 0.1 unit/L *E. coli *RNase-inhibitor at 37°C for 20 minutes. PCR was performed in a 50 μl reaction mixture containing cDNA (derived from 0.5 μg mRNA), 150 nM of each primer, master mix containing Taq polymerase, dNTPs, MgCl_2 _and buffer (5 Prime MasterMix, Cat. No. PRME2200100, VWR, Oslo, Norway), and ultra pure distilled water (Cat. No. 10977-035, Gibco, Invitrogen, Oslo, Norway). The PCR was performed at 94°C for 5 minutes (first denaturation), 94°C for 30 sec (denaturation), 55°C for 30 sec (annealing) and 72°C for 1 minute (extension) for a total of 30 cycles with a 10-minute final extension at 72°C. All reactions were run using a Perkin-Elmer GeneAmpPCR system 2400 (Perkin-Elmer, Cambridge, UK).

The nucleotide sequences of PCR primers for human ChemR23 receptor were 5'-TGG TCT ACA GCA TCG TC-3' (sense) and 5'-ATG GCT GGG GTA GGA AGA GT-3' (antisense), and 917 base pair (bp) fragments were expected [17]. We used two primer sets for human prochemerin. The nucleotide sequences for primer set one were 5'-GAA GAA ACC CGA GTG CAA AG-3' (sense) and 5'-CTT GGA GAA GGC GAA CTG TC-3' (antisense), and 229 bp fragments were expected [18]. The nucleotide sequences for primer set two were 5'-GGA GGA ATT TCA CAA GCA C-3' (sense) and 5'-GAA CTG TCC AGG GAA GTA GA-3'(antisense), and 361 bp fragments were expected [19].

To test the quality of mRNA, the presence of a housekeeping gene transcript, adenine phosphoribosyltransferase (APRT), was assayed. 5'-CCC GAG GCT TCC TCT TTG GC-3' (sense), 5'-CTC CCT GCC CTT AAG CGA GG-3' (antisense), and contaminating DNA would generate a 800-bp fragment, whereas mRNA would generate a 300-bp fragment [16]. Genomic DNA was obtained from DNA isolated from human leukocytes and was used to assess possible contamination.

PCR products were analysed by the use of polyacrylamide gel (Novex TBE gel 6% Cat. No. EC6265BOX, Invitrogen), stained with SYBR-safe DNA gel stain (Cat. No. S33102, Invitrogen) and photographed under UV-light using a G-BOX (Syngene, Cambridge, UK).

The sequences of the amplicons were confirmed using BigDye^® ^Terminator v3.1 Cycle Sequencing Kit (Cat. No. 4337455, Applied Biosystems, Foster City, CA, USA). A total of 2 μl of each PCR product and 1 μM of each primer were processed according to the kit manual. The cycle sequencing was performed on the GeneAmp^® ^PCR Systems 9700 (Applied Biosystems) while the purification was done by capillary gel-electrophoresis on the 3130XL Genetic analyzer (Applied Biosystems).

### Immunocytochemistry

To achieve the required amount of cells for *in vitro *experiments, cells were passaged four times. The phenotype was assessed at the time of experiments by immunolabelling for collagen type II and aggrecan, using the primary antibodies: polyclonal rabbit anti-human collagen II (Cat. No. ab34712, Abcam, Cambridge, UK) and monoclonal mouse anti-human aggrecan (Cat. No. ab3778, Abcam). The secondary antibodies used were: polyclonal goat anti-rabbit IgG conjugated with Alexa Fluor 594 (Cat. No. A21207, Invitrogen) and polyclonal rabbit anti-mouse IgG conjugated with Alexa Fluor 488 (Cat. No. A11059, Invitrogen). Identification of ChemR23 and chemerin was performed with the primary antibodies polyclonal rabbit anti-human ChemR23 antibody (Cat. No. ab13172, Abcam), and polyclonal goat anti-human TIG-2 antibody (Cat. No. sc-47482, Santa Cruz Biotechnology, Heidelberg, Germany). The secondary antibodies used were: goat anti-rabbit IgG conjugated with Alexa Fluor 488 (Cat. No. A11008, Invitrogen), and anti-goat IgG conjugated with Alexa Fluor 594 (Cat. No. A11058, Invitrogen). Chondrocyte cultures were grown on fibronectin coated chamber slides (Cat. No. 154534, Nunc, Roskilde, Denmark) for 24 h (ChemR23 and chemerin labelling), and for seven days (collagen II and aggrecan labelling). The cultures were washed twice with phosphate buffered saline (PBS) and fixed for 10 minutes in cold PBS containing 0.2 M sucrose and 4% paraformaldehyde. After fixation, the slides were blocked for one hour with PBS containing 1% bovine serum albumin (BSA). Thereafter, cell cultures were incubated at 4°C overnight with the primary antibodies. The slides were then washed three times in PBS and incubated with secondary antibodies for one hour in room temperature. Isotype control was used to assess non-specific binding. The slides were mounted by adding DAPI-fluoromount G (Cat. No. 0100-20, SouthernBiotech, Birmingham, AL, USA) and examined in a Zeiss axiophot photomicroscope (Carl Zeiss, Oberkochen, Germany).

### Immunohistochemistry

Immunohistochemical studies were performed to investigate whether ChemR23 and chemerin were present in native cartilage tissue. Biopsies were fixed in paraformaldehyde (4%) containing 0.2 M sucrose in PBS. After 48 h, the tissue was embedded in paraffin and sectioned at 5 μm thickness onto poly-L-Lysine coated slides (0.01%, Sigma Aldrich). Sections were deparaffinised by xylene and graded alcohol washes and immersed in distilled water. Thereafter, sections were incubated in PBS containing 1% BSA for 60 minutes followed by incubation with monoclonal mouse anti-human ChemR23 (Cat. No. MAB362, R&D Systems, Abingdon, UK), diluted at 1:100 and incubated at 4°C overnight. After rinsing in PBS, sections were incubated for 45 minutes with secondary goat anti-mouse antibody conjugated with horseradish peroxidase (SuperPicTure Polymer detection kit, Invitrogen). For the detection of chemerin, polyclonal goat anti-human TIG-2 (Cat. No. sc-47482, Santa Cruz Biotechnology) was used, followed by an Alexa Fluor 594 conjugated donkey anti-goat IgG antibody (Cat. No. A11058, Invitrogen) for detection. Sections were mounted by adding DAPI-fluoromount G (Cat. No. 0100-20, SouthernBiotech). Matched isotype antibodies were used as a control for non-specific background staining.

### Western blotting

Intracellular signal transduction in chondrocytes stimulated with chemerin was investigated by immunoblotting of phosphorylated MAPKs p44/42 (Thr202/Tyr204) and phosphorylated Akt (Ser473). To detect the phosphorylated MAPKs, a phospho-Erk1/2 pathway sampler kit was used (Cat. No. 9911, Cell Signaling Technology, Boston, MA, USA). Phospho-specific antibody towards phospho-Akt (Ser473) was used to detect the ChemR23 mediated phosphorylation of Akt. Cell cultures were treated with 10 nM recombinant human chemerin^21-157 ^(*E*. *coli *derived Glu 21 - Ser 157 with an N-terminal Met, Cat. No. 2324-CM, R&D Systems) at various time points. Cultures added medium only served as controls, and a MEK1/2 inhibitor U0126 (Cat. No. 9900, Invitrogen) was added to some cultures one hour prior to challenge with 10 nM chemerin. A number of 0.5 x10^6 ^cells were seeded per well in a six-multiwell plate (Cat. No. 3046, Falcon, BD Biosciences, Trondheim, Norway) and grown in a culture medium with 10% FCS for 24 h. Subsequently, the cells were washed twice in PBS and grown under reduced-serum conditions (0.1%) for 24 h. Thereafter, cultures were washed twice and challenged with 10 nM chemerin for 1 minute, 2.5 minutes, 5 minutes and 10 minutes. Cells were then harvested directly in 150 μl SDS-buffer containing NuPAGE LDS sample buffer (Cat. No. NP0007, Invitrogen), NuPAGE Reducing agent (Cat. No. NP 0004, Invitrogen), phosphatase inhibitor (Cat. No. 78420, Thermo Scientific, Chicago, IL, USA), protease inhibitor (Cat. No. 04693124001, Roche Applied Science, Basel, Switzerland), and distilled water.

The amount of total protein was measured in each lysate using Modular E 170 (Roche Diagnostics, Mannheim, Germany). The samples were heated to 100°C for five minutes before an equal amount of protein from each extract (390 μg) were loaded into different wells. A total of 15 μl of a pre-stained protein marker (Cat. No. 7720, Cell Signaling Technology) was added to control the efficacy of the electrophoresis. Ten μl of a biotinylated protein ladder (Cat. No. 7727, Cell Signaling Technology) to assess the molecular weights (kDa) of proteins were also added. Proteins were separated by electrophoresis in NuPAGE Mes SDS running buffer (Cat. No. NP 0002, Invitrogen) at 200 V (constant), using 100-125 mA per gel (NuPAGE 4-12% BIS-tris gels, Cat. No. NP0323, Invitrogen) for 35 min. Electroblotting was performed by electrontransfer onto PVDF-membranes (Cat. No. LC2005, Invitrogen) in NuPAGE transfer buffer (Cat. No. NP0006, Invitrogen) with 10% methanol at 30 V (constant), using 170 mA per gel transfer for 1 h. After electroblotting, the membranes were blocked with 5% non-fat dry milk/0.1% Tween 20 for 1 h at room temperature. Next, the membranes were incubated with primary antibodies overnight at 4 °C in 5% BSA/0.1% Tween 20. The phospho-p44/42 (Thr202/Tyr204) antibody was used at a 1:2000 dilution and the phospho-Akt (Ser473) antibody was diluted at 1:1000. To control for equal loading amounts the membranes were incubated with β-actin antibody (Cat. No. 4970, Cell Signaling Technology), dilution 1:1000. The membranes were then washed and incubated with horseradish peroxidase (HRP)-conjugated goat anti-rabbit IgG (Cat. No. 7074, Cell Signaling Technology) and HRP-conjugated anti-biotin antibody (Cat. No. 7075, Cell Signaling Technology) for 1 h at room temperature. Blots were detected by adding substrate containing Lumiglo reagent and peroxide (Cat. No. 7003, Cell Signaling Technology) and developed with Fujifilm LAS-3000. A densitometric comparison between the protein bands was performed using the GeneTools software (Syngene, Cambridge, UK).

### Cytokine and metalloprotease measurements

Chondrocyte cultures (0.5 × 10^6 ^cells/well) were incubated for 24 h in medium supplemented with 10% serum. Then, the cells were washed twice with PBS and further incubated for 24 h and grown under reduced-serum conditions (0.1%). The cultures were then washed twice and one culture received medium with 10 nM chemerin^21-157^, another received medium with 100 nM chemerin^21-157 ^and a third was added a medium with vehicle only as control. The cultures were incubated for 24 h before the medium supernatants were aliqouted and frozen in -70°C for later analysis.

Cytokines were measured with a suspension array analytical platform (Bio-Plex 200, Bio-Rad, Hercules, CA, USA). One ampoule of each supernatant was thawed on ice, and the amount of total protein was measured in each supernatant using a protein assay kit (Cat. No. 23227, Thermo Scientific, Rockford, IL, USA) before levels of TNF-α, IL-1β, IL-6 and IL-8 were measured using a 4-plex cytokine assay (Cat. No. X50053UVBS, Bio-Rad). The samples were run in a 1:4 dilution in duplicates.

Likewise, a multiplex MMP-assay was used to measure the levels of the metalloproteases MMP-1, -2, -3, -7, -8, -9, -12 and -13 (Cat. No. LMP000, R&D Systems) using a Bio-Plex 200 analyser. The samples were run in a 1:4 dilution in duplicates.

### Statistical analysis

Data were analysed using SPSS statistical software version 16.0 (SPSS Inc., Chicago, IL, USA). Cytokines and metalloproteases were examined for statistical significance using the Wilcoxon signed-rank test. All data are expressed as mean ± standard error of the mean (SEM). A *P*-value less than 0.05 denoted the presence of a statistically significant difference.

## Results

### Chondrocyte cultures

**Figure 1:**
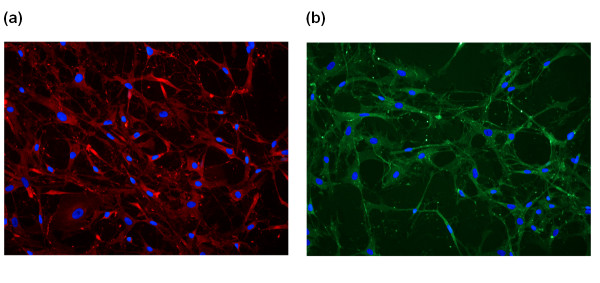
**Expression of collagen type II and aggrecan in cultured human articular chondrocytes**. **(a) **Cells were labelled with polyclonal rabbit anti-human collagen type II and secondary antibody conjugated with Alexa Fluor 594 (red). **(b) **Cells were labelled with monoclonal mouse anti-human aggrecan and secondary antibody conjugated with Alexa Fluor 488 (green). The nuclei were visualized by Dapi dye (blue). Isotype controls had no staining (not shown).

It has been demonstrated that chondrocytes gradually lose their chondrogenic properties during serial passage in monolayer [20,21]. To ensure a chondrogenic phenotype, cells were immunolabelled for aggrecan and collagen type II after propagation in culture corresponding to the time preceding *in vitro *experiments. As judged by these parameters, the chondrogenic phenotype was preserved (Figure 1).

### ChemR23 and chemerin expression in human articular chondrocytes

#### ChemR23 expression by RT-PCR

**Figure 2:**
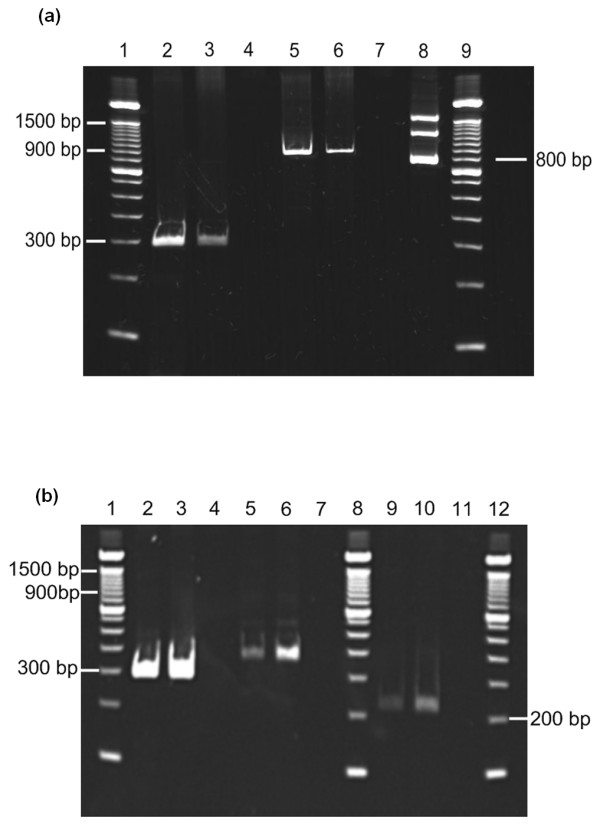
**Expression of ChemR23 and chemerin in cultured human articular chondrocytes as detected by RT-PCR**. **(a) **Lanes 2 and 3: RT-PCR with APRT primers and cDNA from two individual cell cultures. Lane 4: APRT primers without cDNA. Lanes 5 and 6: ChemR23 primers and cDNA from two individual cell cultures. Lane 7: ChemR23 primers without cDNA. Lane 8: APRT primers and genomic DNA. **(b) **Lanes 2 and 3: RT-PCR with APRT primers and cDNA from two individual cell cultures. Lane 4: APRT primers without cDNA. Lanes 5 and 6: Prochemerin primer pair 1 and cDNA from two individual cell cultures. Lane 7: Prochemerin primers without cDNA. Lanes 9 and 10: Prochemerin primer pair 2 and cDNA from two individual cell cultures. Lane 11: Prochemerin primers without cDNA.

To clarify whether cultured human chondrocytes express ChemR23, mRNA isolated from six different cell cultures were analysed for ChemR23 transcripts by RT-PCR. Figure 2a shows the ChemR23 transcripts in chondrocyte cultures from two patients subjected to total knee arthroplasty due to severe osteoarthritis. The PCR products detected by gel electrophoresis revealed that mRNA corresponding to the 917 bp transcript of the ChemR23 was present (Figure 2a, Lanes 5 and 6). The APRT primers were designed to give an 800 bp band in case of contamination with genomic DNA, whereas the presence of a 300 bp band would correspond to the mRNA transcript for the APRT gene. As shown in the figure, genomic DNA was not detected and both controls (Lanes 4 and 7) were negative. The 917 bp transcript was identified in all the tested cultures: three patients subjected to ACT due to cartilage lesion and another three patients suffering from severe osteoarthritis. Sequencing of the PCR products confirmed that they were transcripts for ChemR23 and APRT as judged by information obtained from the GeneBank (NCBI) (Data not shown).

#### Chemerin expression by RT-PCR

To detect the presence of chemerin in chondrocytes, mRNA isolated from two individual cell cultures was analysed for prochemerin transcripts using two different primer sets. Figure 2b shows prochemerin transcripts in chondrocyte cultures from two patients subjected to a total knee arthroplasty. The PCR products detected by gel electrophoresis showed that mRNA corresponding to the 229 bp transcript and the 361 bp transcript of prochemerin was present in both cultures (Figure 2b, Lanes 5, 6 and 9, 10). Genomic DNA was not detected (Lanes 2 and 3) and all controls (Lanes 4, 7 and 11) were negative. Sequencing of the PCR products confirmed that they were transcripts for chemerin and APRT as judged by information obtained from the GeneBank (NCBI) (Data not shown).

#### ChemR23 and chemerin expression in native cartilage

**Figure 3:**
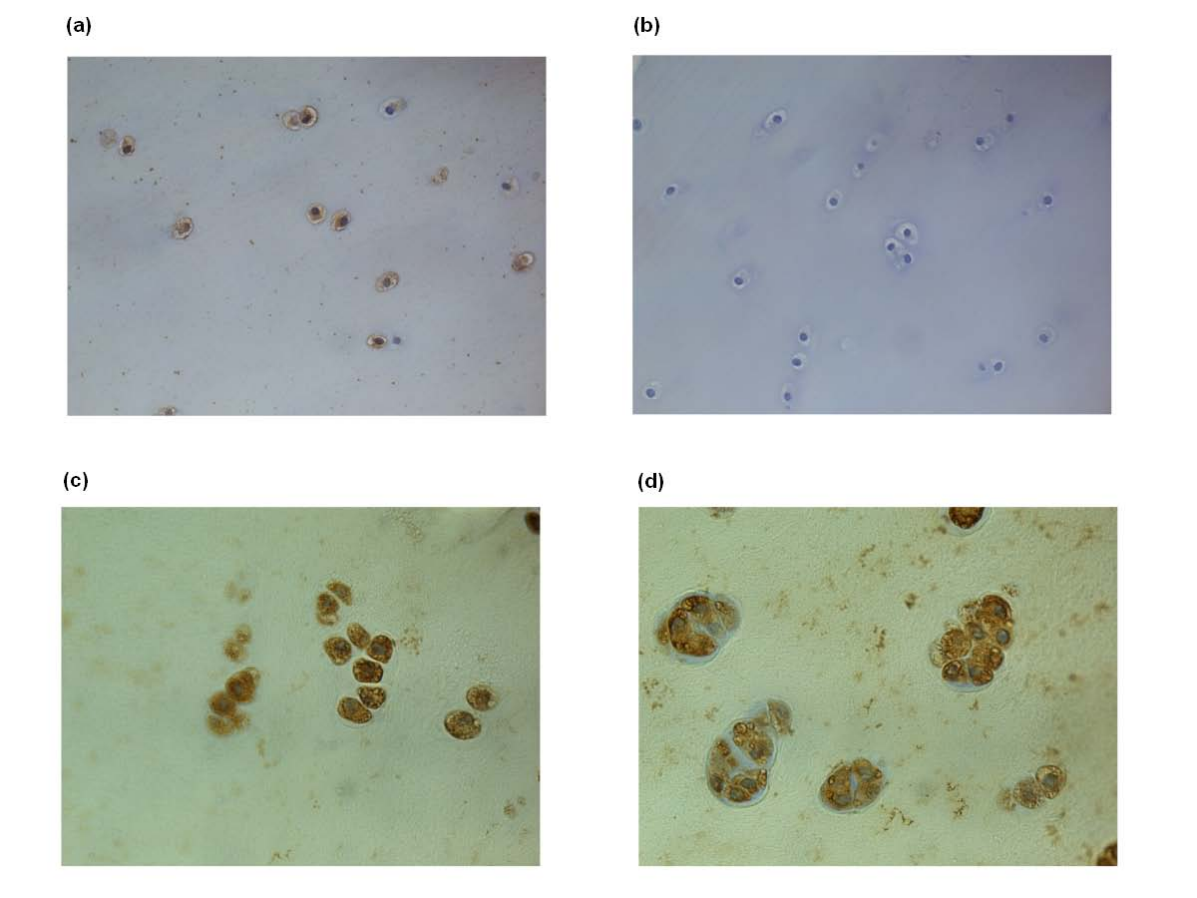
**The presence of ChemR23 in sections of human articular cartilage**. **(a) **Micrograph A (20X) shows positively (brown) stained chondrocytes in tissue from one patient subjected to total knee arthroplasty. **(b) **Micrograph B shows an isotype control which was negative. **(c and d) **Micrograph C (40X) and D (60X) shows positively stained chondrocytes from one patient undergoing reconstruction of ligament.

**Figure 4:**
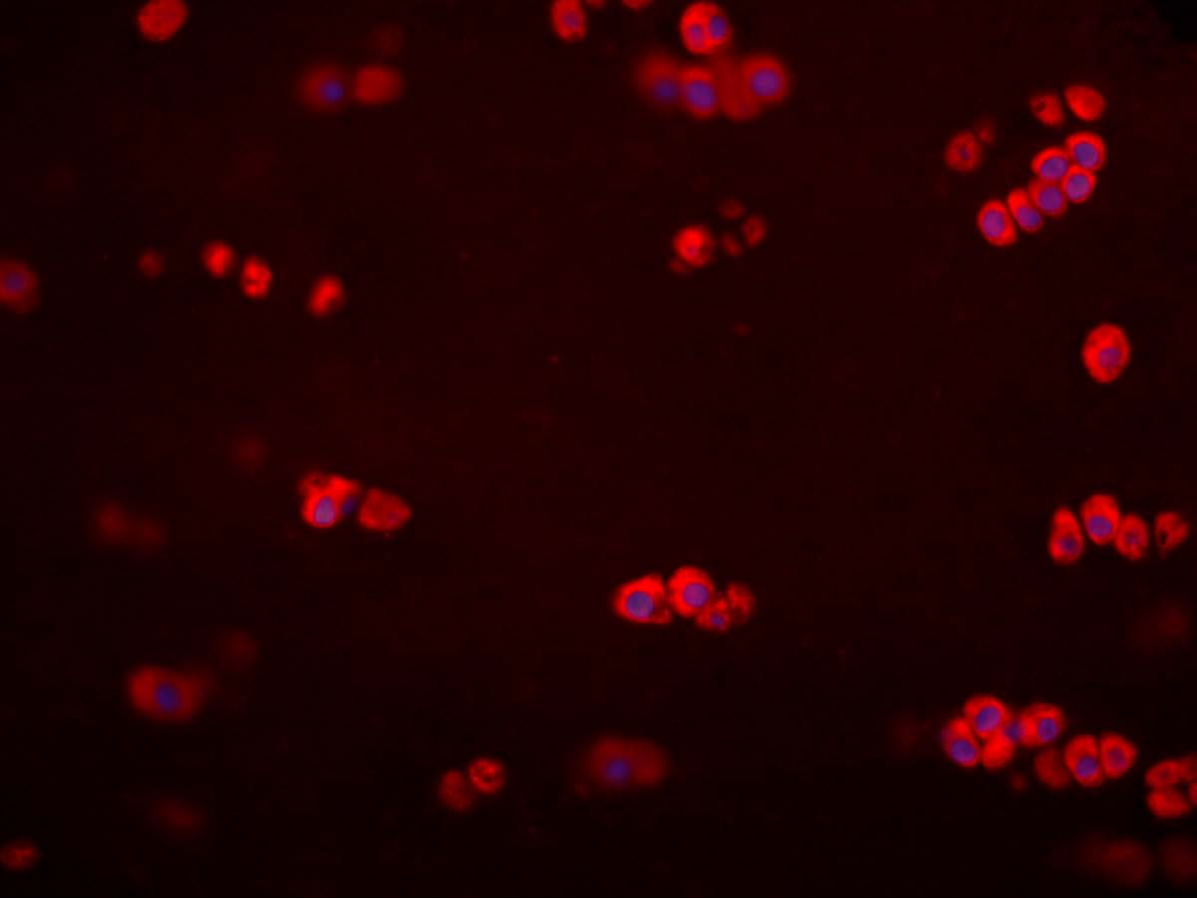
**The presence of chemerin in sections of human articular cartilage**. The micrograph (40X) shows positively (red) stained chondrocytes in tissue from one patient undergoing ligament repair. Nuclei were visualized by Dapi dye (blue). Negative controls (isotype IgG) had no red staining (not shown).

The presence of ChemR23 and chemerin proteins in native cartilage was investigated by immunohistochemistry. Cartilage biopsies from two patients subjected to ACT, four patients subjected to total knee arthroplasty and three patients undergoing reconstruction of ligaments were used. In all cases, cells residing in cartilage tissue were positively stained for both ChemR23 (Figure 3) and chemerin (Figure 4).

#### ChemR23 and chemerin expression in vitro

**Figure 5:**
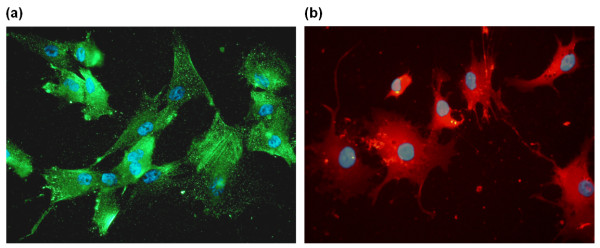
**The presence of ChemR23 and chemerin in cultured human articular chondrocytes**. **(a) **Cells were labelled with polyclonal rabbit anti-human ChemR23 and secondary antibody conjugated with Alexa Fluor 488 (green). **(b) **Cells were labelled with polyclonal goat anti-human TIG-2 (chemerin) and secondary antibody conjugated with Alexa Fluor 594 (red). Nuclei were visualized by Dapi dye (blue). Isotype controls had no staining (not shown).

The presence of ChemR23 and chemerin was investigated by immunocytochemistry of chondrocyte cultures established from biopsies taken from seven individual patients, three that were subjected to ACT, another three subjected to total knee arthroplasty and one undergoing reconstruction of a ligament. In all cases, cells were positively stained for both ChemR23 and chemerin (Figure 5)

#### Chemerin^21-157 ^stimulated the phosphorylation of MAPKs and Akt

**Figure 6:**
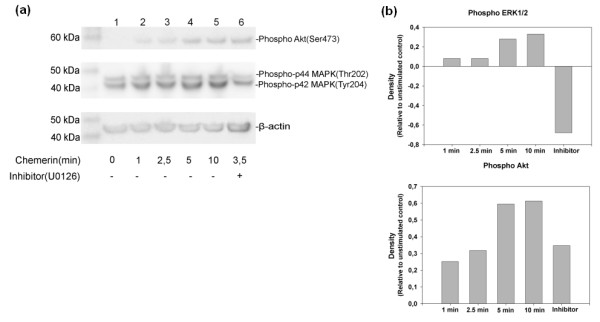
**Western blot of phosphorylated p44/42 MAPKs (Thr202/Tyr204) and phosphorylated Akt (Ser 473)**. **(a) **Cultured chondrocytes were challenged with 10 nM chemerin^21-157 ^for 1, 2.5, 5 and 10 minutes. Lane 1 represents the control where no chemerin was added and Lane 6 represents the sample extract where the MEK 1/2 kinase inhibitor U0126 was added 1 h prior to a 3.5 minutes chemerin^21-157 ^challenge. **(b) **The density of each band was normalized to β-actin, the graphs shows the increase in density relative to unstimulated control.

To assess whether intracellular signalling pathways were engaged upon ligand-receptor binding, Western blots of phospho-p44/42 MAPKs and phospho-Akt (Ser 473) were performed. In separate experiments, cultured chondrocytes from three patients subjected to total knee arthroplasty were challenged with 10 nM chemerin^21-157 ^for 1 minute, 2.5 minutes, 5 minutes and 10 minutes, respectively. Figure 6 shows that both p44/42 MAPKs and Akt (Ser 473) were phosphorylated at specific residues. Challenging with chemerin^21-157 ^for 5 and 10 minutes showed a markedly increased phosphorylation of the p44/42 MAPKs compared to the unstimulated control, and inhibiting the MEK 1/2 pathway led to a reduction of phosphorylated p44/42 MAPK including an inhibition of the background phosphorylated p44/42 MAPK, as shown by a negative density value compared to the unstimulated control (Figure 6b).

Phosho-Akt levels increased from 1 minute up to 10 minutes after stimulation with chemerin^21-157 ^relative to the control. These results demonstrate that chemerin^21-157 ^binding to ChemR23 increases phosphorylation of Akt which may induce activation of MEK1/2 and further activate the MAPK pathway. Furthermore, addition of the MEK 1/2 inhibitor did not affect the activation of phospho-Akt after stimulation with chemerin^21-157 ^for 3.5 minutes.

#### Chemerin^21-157 ^promoted the secretion of pro-inflammatory cytokines and MMPs

**Figure 7:**
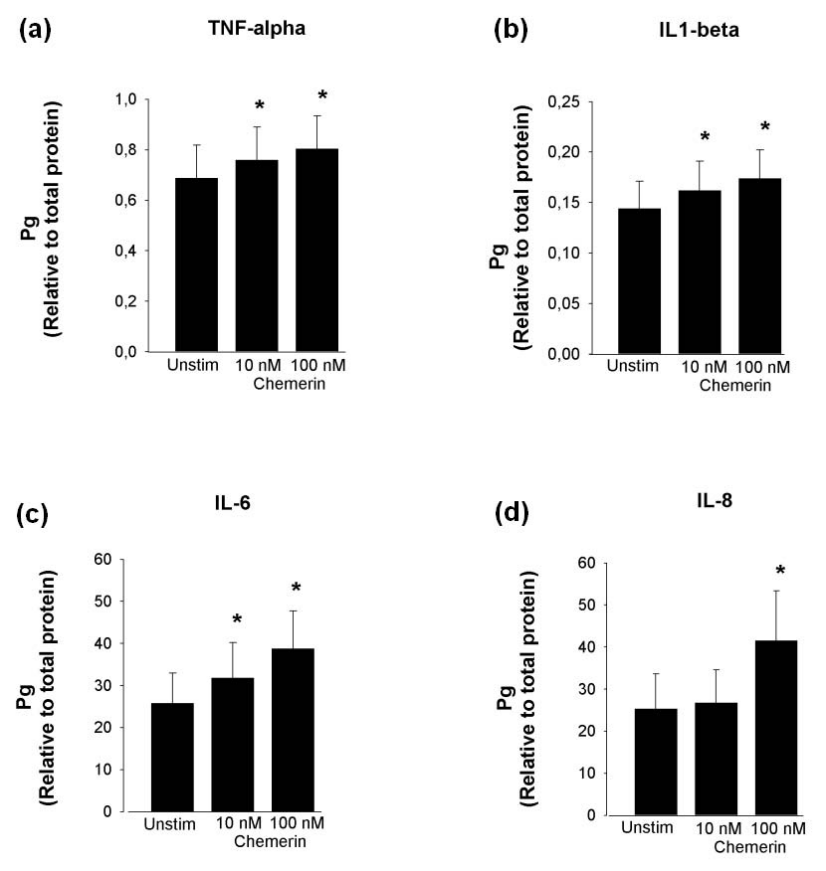
**Cytokine levels in supernatants from human articular chondrocytes stimulated with recombinant human chemerin**^**21-157**^. Cell supernatants were assessed for cytokine contents after 24 h of stimulation with 10 nM or 100 nM recombinant chemerin^21-157^. The levels of TNF-α **(a)**, IL-1β **(b)**, IL-6 **(c)**, and IL-8 **(d) **are shown as mean ± standard error of the mean. Results are from six separate experiments analyzed in duplicates. Concentration is given relative to amount of protein (μg/ml). *P < 0.05, stimulated versus unstimulated.

Based on the findings that ChemR23 expressed by chondrocytes transduced intracellular signalling in the presence of recombinant chemerin^21-157^, further studies were conducted to investigate the biological significance. Chondrocytes from three patients subjected to ACT, and another three individuals subjected to total knee arthroplasty, were in separate experiments challenged with 10 nM or 100 nM chemerin^21-157 ^for 24 h, and subsequently a panel of cytokines was measured in the cell supernatants. The results (Figure 7) show an increased concentration of TNF-α, IL-1β, IL-6 and IL-8 as a result of chemerin stimulation in comparison to unstimulated control cells (*P *< 0.05). The levels of IL-6 and IL-8 were markedly increased, whereas a rather modest effect was observed in terms of altered levels of IL-1β and TNF-α.

**Figure 8:**
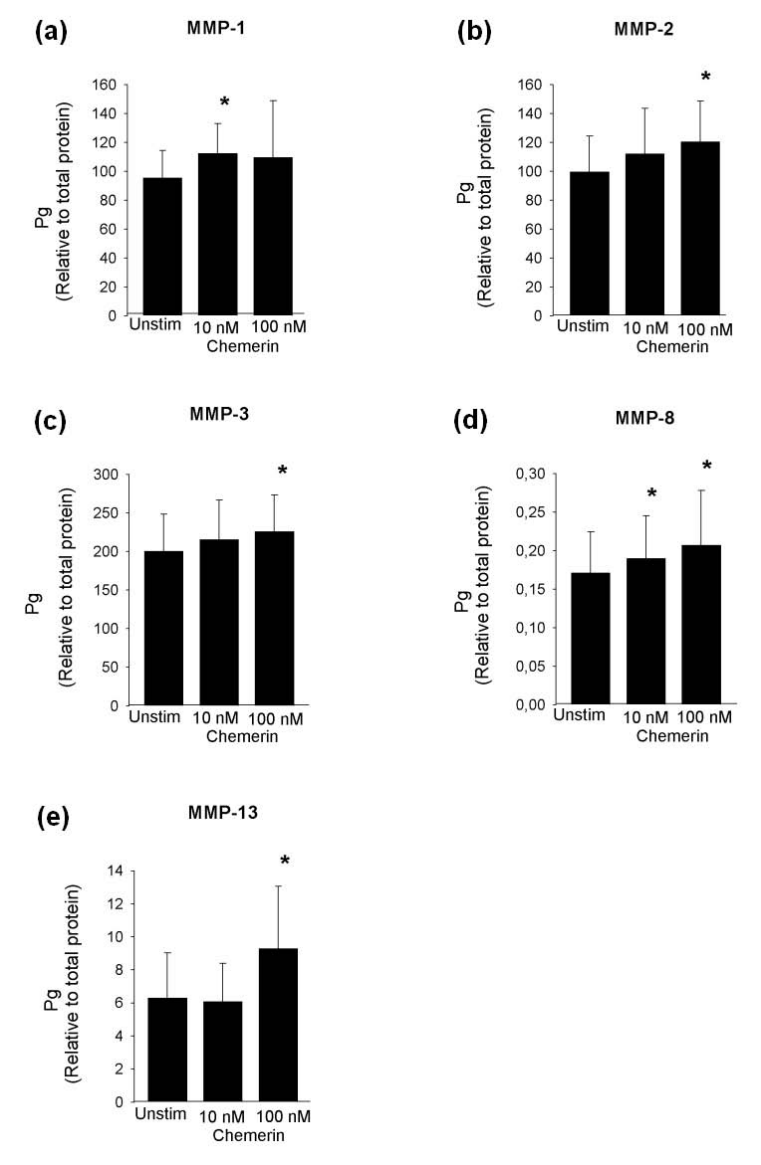
**MMP levels in supernatants from human articular chondrocytes stimulated with recombinant human chemerin**^**21-157**^. Cell supernatants were assessed for content of MMPs after 24 h of stimulation with 10 nM or 100 nM recombinant chemerin^21-157^. The levels of MMP-1 **(a)**, MMP-2 **(b)**, MMP-3 **(c)**, MMP-8 **(d)**, and MMP-13 **(e) **are shown as mean ± standard error of the mean values. Results are from six separate experiments analyzed in duplicates. Concentration is given relative to amount of protein (μg/ml). *P < 0.05, stimulated versus unstimulated.

Joint inflammation is associated with deterioration of the cartilage matrix requiring a clarification as to whether chemerin^21-157 ^affects chondrocyte secretion of matrix metalloproteases. Cell cultures from six individuals were arranged and challenged with 10 nM or 100 nM chemerin^21-157 ^for 24 h, and subsequently a panel of eight different MMPs was measured in the supernatants. Significantly (*P *< 0.05) elevated levels of MMP-1, MMP-2, MMP-3, MMP-8, and MMP-13 were detected (Figure 8). The metalloproteases MMP-7, MMP-9, and MMP-12 could not be detected.

## Discussion

Recent studies addressing the role of chondrocytes in joint inflammation have revealed that these cells secrete and bind a variety of cytokines and chemokines [22-25] and that they possess immunoregulatory capabilities [26]. The present study adds further information to this issue by demonstrating that chondrocytes in both native cartilage and cell culture express the chemokine receptor ChemR23, a property primarily ascribed to leukocytes.

Using the ligand recombinant human chemerin^21-157^, we demonstrated that chemerin/ChemR23 binding elicits intracellular signalling leading to the phosphorylation of p44/42 MAPKs and Akt (Ser 473), both of which are involved in central signal-transduction pathways that convey inflammatory signalling [27,28]. Hence, the cleavage product of prochemerin chemerin^21-157 ^mediates pro-inflammatory signalling in chondrocytes as judged by the observed promotion of cytokine secretion.

The enzymes reported to generate chemerin^21-157 ^from prochemerin include the neutrophil serine proteases cathepsin G and elastase [1]. This indicates that, regardless of the source of prochemerin in joints, it can be cleaved by the enzymes produced by neutrophils into isoforms of chemerin that further promote inflammation by recruiting leukocytes, and that promote chondrocyte secretion of pro-inflammatory cytokines. Previous studies have reported that chemerin^21-157 ^can be detected in arthritic synovial fluid [4,5] and prochemerin from the circulation could likely be the source. However, in the present study both cultured chondrocytes and cells in native tissue were immunopositive towards chemerin. Taken together with the finding that also mRNA for prochemerin was present in chondrocytes, this strongly suggests that these cells produce prochemerin that may serve as substrate for neutrophil-derived serine proteases to generate chemerin^21-157^. Hence, resident chondrocytes secrete a chemokine precursor that, after enzymatic cleavage by enzymes secreted by neutrophils, further may recruit leukocytes expressing the ChemR23 receptor. In addition, the cleavage product chemerin^21-157 ^can bind the ChemR23 receptor expressed by chondrocytes which promote their secretion of pro-inflammatory cytokines and MMPs.

A marked elevation of IL-8 and IL-6 was observed as a result of chemerin^21-157 ^stimulation, whereas TNF-α and IL-1β were modestly altered. Nevertheless, despite low levels these may be sufficient to orchestrate an inflammatory process due to their strong synergistic effects, even at low concentrations [29]. In contrast, there is a rather indisputably strong association between the content of TNF-α in synovial fluid and disease activity such as in rheumatoid arthritis [30]. In our study, the sole cytokine source was the chondrocytes, unlike the situation occurring in a diseased joint where leukocytes are also present. Yet according to previous reports, the production of IL-6, IL-8 and MMPs in chondrocytes is assigned to the action of TNF-α and IL-1β [25,31-34]. However, chemerin^21-157 ^may have induced an immediate release of TNF-α and IL-1β followed by internalization and degradation, whereas IL-6, IL-8 and MMPs rely on the autocrine action of TNF-α and IL-1β as reflected at the time of measurement.

IL-8 exerts a potent chemotactic activity towards neutrophils [35], whereby it has a decisive role in the initial stages of inflammation. Even so, the present study indicates that chemerin may be a prerequisite for an augmented secretion of IL-8. Consequently, chemerin/ChemR23 could serve as a central link for the initiation and maintenance of inflammation in joints.

It has previously been described that chondrocytes produce IL-6 in response to physiologic and inflammatory stimuli, and that IL-6 may serve as a mediator coordinating responses to cartilage injury [32]. Since IL-6 modulates the growth and differentiation of B- and T-lymphocytes [36,37], our findings propose that chemerin/ChemR23 signalling may contribute to the activation of B- and T-cells leading to engagement of adaptive immunity and further maturation of inflammation in joints.

MMP-2, MMP-3 and MMP-13 cleave the most abundant proteoglycan in cartilage, aggrecan, at the Asn^373^-Phe^342 ^bond, and the resulting major fragment can be detected in the synovial fluid from patients with various arthritic diseases [38]. It has previously been reported that these MMPs are produced by chondrocytes *in vitro *[39-43]. The present results showed that chemerin^21-157 ^stimulation significantly increased secretion of MMP-1, MMP-2, MMP-3, MMP-8 and MMP-13. This indicates that chemerin^21-157 ^promotes secretion of enzymes that digest the extracellular matrix, leading to deterioration of cartilage tissue.

It was not our aim to compare the effect of chemerin^21-157 ^on chondrocytes from healthy and diseased joints, it appeared, however, that cells from the healthiest donors (ACT) secreted lower amounts of cytokines than OA cells. Using 100 nM chemerin^21-157^, the elevated secretion of IL-8 compared to unstimulated control was markedly lower (8 pg/ml) for ACT cells compared to OA cells (25 pg/ml). This warrants a further investigation of the effect of chemerin on chondrocytes in diseased and healthy stages.

## Conclusions

We demonstrate that human articular chondrocytes express the chemoattractant receptor ChemR23 and its ligand chemerin. The latter being a chemokine that directs migration of ChemR23^+ ^leukocytes. In chondrocytes, the isoform chemerin^21-157 ^activates the intracellular signalling cascades MAPKs and Akt, followed by an enhanced secretion of pro-inflammatory cytokines and MMPs. This implies that chemerin/ChemR23 signalling in chondrocytes is capable of recruiting leukocytes to inflamed joints, and that this signalling also can mediate cartilage deterioration. In view of the inflammatory properties of chemerin/ChemR23, this study reveals a molecular signalling mechanism which may be targeted by appropriate inhibitors to reduce joint inflammation and cartilage degradation.

## Abbreviations

ACT: autologous chondrocyte transplantation; APRT: adenine phosphoribosyltransferase; BSA: bovine serum albumin; CMKLR1: chemokine-like receptor 1; DC: dendritic cell; EPA: eicosapentaenoic acid; FCS: foetal calf serum; HRP: horseradish peroxidase; MAPK: mitogen activated protein kinase; MMP: matrix metallo proteases; OA: osteoarthritis; PBS: phosphate buffered saline; RT-PCR: reverse transcriptase polymerase chain reaction; RvE1: resolvinE1; TIG 2: tazarotene-induced gene 2.

## Competing interests

The authors declare that they have no competing interests.

## Authors' contributions

VB performed all the analyses except for the immunohistochemistry, participated in the study design and coordination, and in the drafting of the manuscript. BS participated in the design and coordination, helped to draft the manuscript, performed the immunohistochemistry and revised the manuscript. SB participated in cell cultivation, data acquisition and in the revision of the manuscript. JB participated in the study design and coordination, in the interpretation of results, and in the revision of the manuscript. KM participated in the acquisition of biopsies, interpretation of the results and in the revision of the manuscript. YF participated in the study design and coordination, helped to draft the manuscript, interpret results, and revised the manuscript. All the authors gave their final approval of the manuscript version to be published.
